# Sarcopenia is Predictive of Functional Outcomes in Older Trauma Patients

**DOI:** 10.7759/cureus.6154

**Published:** 2019-11-14

**Authors:** Franchesca Hwang, Christopher M McGreevy, Sri Ram Pentakota, Davis Verde, Joo Hye Park, Ana Berlin, Nina E Glass, David H Livingston, Anne Mosenthal

**Affiliations:** 1 General Surgery, Rutgers New Jersey Medical School, Newark, USA; 2 Breast Surgery, University of Pennsylvania, Philadelphia, USA; 3 Surgery, Rutgers New Jersey Medical School, Newark, USA; 4 Anesthesiology, Columbia University, New York, USA; 5 Internal Medicine, Keck School of Medicine of the University of Southern California, Los Angeles, USA; 6 Surgery, Columbia University, New York, USA

**Keywords:** geriatric trauma, trauma icu, sarcopenia, mortality, functional outcomes, glasgow outcome scale, older trauma patients

## Abstract

Introduction: Older patients are more vulnerable to poor outcomes after trauma than younger patients. Sarcopenia, loss of skeletal mass, is prevalent in trauma patients admitted to the intensive care unit (ICU), and it has been shown to correlate with adverse outcomes, such as mortality and ICU days. Yet, little is known whether it predicts other outcomes. We hypothesized that sarcopenia independently predicts poor functional outcomes in older trauma patients admitted to the ICU.

Methods: We performed a retrospective review of patients aged >55 admitted to a surgical ICU in a Level I trauma center for two years. Sarcopenic status was determined by measuring total skeletal muscle cross-sectional area at the L3 level on admission computed tomography (CT), normalized for height with sex-specific cutoffs. Primary outcome measures were in-hospital mortality, functional outcomes measured by the Glasgow Outcome Scale (GOS) at discharge, and discharge disposition. Multivariable logistic regression was used to determine predictors of primary outcomes.

Results: Out of 230 patients, 32% were sarcopenic. The overall mortality was 20%, and 30% were discharged with poor functional outcomes. A higher proportion of sarcopenic patients among survivors had poor functional outcomes at discharge (55% vs. 30%, p=0.002). Sarcopenia was not predictive of in-hospital mortality but was an independent predictor of poor functional outcomes at discharge (OR 2.6; 95% confidence interval [CI] 1.3-5.5), adjusting for age, Glasgow Coma Scale (GCS) on admission, diagnosis of traumatic brain injury (TBI), Injury Severity Score (ISS), and the number of life-limiting illnesses.

Conclusions: Sarcopenia is prevalent in geriatric trauma ICU patients and is an independent predictor of poor functional outcomes. Assessing for sarcopenia has an important potential as a prognostic tool in older trauma patients.

## Introduction

Older patients are the fastest growing demographic group treated at trauma centers in the United States. It has been well established that they are more vulnerable to poor outcomes, with the highest case fatality rates observed in patients aged 75 years and older [1]. They also have an increased risk for mortality compared to their younger counterparts despite the equivalent injury severity [2-3]. However, age alone does not account for the increase in morbidity and mortality. Several factors have been found to be associated with their higher susceptibility to poor outcomes, including comorbidities, pre-injury functional status, and nutritional state. Frailty has been proposed to be a superior predictor to age alone for adverse outcomes among older trauma patients [4]. However, using frailty for risk stratification in a trauma patient population is quite challenging; many of the frailty indices are complicated and difficult to apply expeditiously at the bedside of an injured patient. As an alternative, sarcopenia has been suggested as a surrogate marker for frailty and has been shown to be an independent predictor of poor in-hospital complications in older trauma patients [5].

Sarcopenia is defined as the loss of skeletal muscle mass and describes a universal effect of aging accompanied by functional, metabolic, and immunologic consequences. In addition to its critical role in mobility, the skeletal muscle maintains protein synthetic rates in other vital tissues during periods of stress as it is the largest reserve of protein in a body [6]. It is also responsible for various immunologic functions, such as antibody production, wound healing, and white blood cell production during an illness [7]. An advantage to using sarcopenia as a marker of frailty is that it can be rapidly and objectively determined from axial computed tomography (CT) imaging, which is routinely performed on trauma patients [8]. Sarcopenia is extremely common and underappreciated in older trauma patients with an incidence of up to 70% in those admitted to the trauma intensive care unit (ICU) [9]. Although it has been shown to correlate with mortality, ICU length of stay, and ventilator days, little is known about how it may correlate with functional outcomes at discharge [2,9].

The objectives of our study were threefold: 1) to examine the prevalence of sarcopenia in older trauma patients admitted to the ICU; 2) to describe patients’ outcomes in hospital and at the time of discharge; and 3) to determine if sarcopenia is an independent predictor of poor functional outcomes in older trauma patients.

## Materials and methods

Data source and study population

This is a retrospective study of trauma patients aged 55 years or older admitted to the surgical ICU at an urban Level I trauma center with a convenience sample of all patients for two years (2012 and 2014). The age of 55 years or older was used as the inclusion criteria based on the evidence in trauma literature suggesting that the mortality rate increases after the age of 55 [10]. We only included patients admitted to the surgical ICU to focus on moderately to severely injured patients to study the effect of sarcopenia as its effect is unlikely to be observed in mildly injured patients admitted to regular wards. Patients were excluded if they did not undergo CT of the abdomen at admission.

Determination of sarcopenic status

A total skeletal muscle cross-sectional area at the third lumbar vertebra (L3) was measured from the admission CT by trained research staff. Muscle index, which is normalized muscle mass for height, was subsequently calculated. The presence of sarcopenia was determined using established sex-specific cutoffs for muscle index (<55.4 cm^2^/m^2^ for men and <38.9 cm^2^/m^2^ for women) [11].

Study variables

Data collected included patient demographics, mechanisms of injury, Injury Severity Score (ISS), Glasgow Coma Scale (GCS) at presentation, and comorbidities. Specific comorbidities collected were life-limiting illnesses: end-stage renal disease on hemodialysis, end-stage liver disease, advanced dementia, chronic obstructive pulmonary disease (COPD) requiring home oxygen, metastatic cancer, history of stroke, or immunosuppressant use. The primary outcome measures were in-hospital mortality, functional status at discharge as measured by the Glasgow Outcome Scale (GOS), and discharge disposition.

GOS, the most widely accepted scale for assessing outcomes after head injury, was used to categorize patients into those who died during the hospitalization (GOS of 1) and those who survived to discharge with either poor or good functional outcomes. GOS of 2 or 3, persistent vegetative state or severe disability, respectively, were categorized as poor functional outcomes for the analysis. GOS of 4 or 5, moderate disability or good recovery, respectively, were considered good functional outcomes. These functional outcomes were determined retrospectively based on assessments by physical or occupational therapists or nurses. Discharge disposition was categorized into two groups: discharge to hospice, long-term acute care (LTAC), or skilled nursing facility (SNF) was defined as discharge to dependent care while discharge to home or acute rehabilitation centers was considered an independent discharge status.

Chi-squared (χ^2^) tests were used to determine the association between sarcopenia and primary outcomes, and multivariable logistic regression models were fit to determine independent predictors of outcomes. All analyses were performed using SAS v.9.4 (SAS Institute, Cary, NC) statistical software.

## Results

Out of 269 patients aged 55 or older and admitted to the surgical ICU during the two years, 230 patients had CT scans at admission, meeting our inclusion criteria. Patient characteristics are shown in Table 1.Most of the patients were male and white. Approximately half of the patients experienced fall as the mechanism of the injury. Most of them (81%) had mild traumatic brain injury (TBI) with GCS of 13-15. Almost 30% of the cohort had one or more life-limiting illnesses.

**Table 1:** Patient characteristics of the cohort (n=230) and by the sarcopenic status *Other mechanisms of injury include bicycle accidents, motorcycle collisions, gunshot wounds, and stab wounds. **Life-limiting illnesses include end-stage renal disease on hemodialysis, end-stage liver disease, advanced dementia, chronic obstructive pulmonary disease (COPD), metastatic cancer, history of stroke, or taking immunosuppressant.

	Cohort n=230	Sarcopenic n= 73 (32%)	Non-Sarcopenic n=157 (68%)
	N (%)	N (%)	N (%)
Age			
55-64	64 (28)	11 (17)	53 (83)
65-74	51 (22)	15 (29)	36 (71)
75-84	54 (23)	18 (33)	36 (67)
>85	61 (27)	29 (48)	32 (52)
Gender			
Male	129 (59)	37 (27)	98 (73)
Female	101 (41)	36 (38)	59 (62)
Race/ethnicity			
White	136 (59)	50 (37)	86 (55)
Black	47 (20)	12 (26)	35 (22)
Other	47 (20)	11 (23)	36 (23)
Mechanisms of Injury			
Fall	122 (53)	46 (38)	76 (62)
Motor vehicle collision	46 (20)	14 (30)	32 (70)
Pedestrian struck	31 (13)	8 (26)	23 (74)
Assault	11 (5)	3 (27)	8 (73)
Other*	20 (9)	2 (10)	18 (90)
Glasgow Coma Scale (GCS)			
13-15	187 (81)	59 (32)	128 (68)
9-12	13 (6)	4 (31)	9 (69)
<8	30 (13)	10 (33)	20 (67)
# of Life-limiting Illnesses**			
0	172 (75)	55 (32)	117 (68)
1	51 (22)	16 (31)	35 (69)
2	7 (3)	2 (29)	5 (71)

A third of the cohort was sarcopenic (32%). An increasing trend of sarcopenia was observed in older patients; almost half of the patients older than 85 were sarcopenic (48%). Among those patients who had a fall as the mechanism of injury, 38% were sarcopenic, which was slightly higher than the percentage of sarcopenic patients in other mechanism of injury groups.

**Figure 1:**
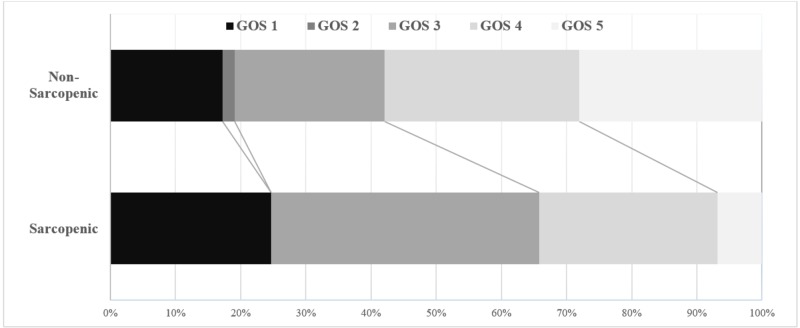
Stacked bar chart of Glasgow Outcome Scale (GOS) distribution by the sarcopenic status

The overall in-hospital mortality rate in the cohort was 20% (n=45) while 30% (n=69) of the cohort were discharged with poor functional outcomes, defined as GOS of 2 or 3 (Table 2). Among those who survived the hospitalization (n=185), most of them were discharged to either home or rehabilitation centers while 6.5% (n=12) were discharged to dependent care (hospice, long-term acute care, or skilled nursing facilities). Stratifying the cohort by their sarcopenic status, a quarter of the sarcopenic patients died in hospital compared to 18% of the non-sarcopenic group (p=0.184). Similarly, a higher proportion of survivors with sarcopenia had poor functional outcomes at discharge (55% vs. 30% non-sarcopenic; p=0.002). Figure 1 demonstrates the distribution of the functional outcomes in sarcopenic and non-sarcopenic patients. Overall, two thirds (66%) of the sarcopenic patients either died or had poor functional outcomes at discharge. Additionally, only 7% of sarcopenic patients had good recovery (GOS of 5) compared to 28% of the sarcopenic group. This difference of functional outcomes among sarcopenic versus non-sarcopenic group reached a statistical significance using a χ2 test, except for in-hospital mortality (Table 2). However, due to the small number of patients (n=12) who were discharged to dependent care, there was no statistically significant association between sarcopenic status and discharge disposition.

**Table 2:** Mortality, functional outcomes, and discharge disposition by the sarcopenic status *Discharge to dependent care defined as discharge to hospice, long-term acute care (LTAC), or skilled nursing facility (SNF). GOS: Glasgow Outcome Scale.

	Sarcopenic (n=73)	Non-Sarcopenic (n=157)	P-values
Mortality (GOS 1)	18 (25%)	27 (18%)	0.184
Functional outcomes among survivors (n=185)			
Poor Functional Outcome (GOS 2 or 3)	30 (55%)	39 (30%)	0.002
Good Functional Outcome (GOS 4 or 5)	25 (45%)	91 (70%)	
Discharge disposition among survivors (n=185)			
Discharge to dependent care*	6 (11%)	6 (5%)	0.11
Acute Rehabilitation Centers/Home	49 (89%)	124 (95%)	

Table 3shows the results of multivariable regression analysis of predictors of the primary outcomes. Although sarcopenia was not a statistically significant predictor of mortality, it was found to be an independent predictor of poor functional outcome at discharge (p=0.011). The odds of poor functional outcomes for survivors is 2.6 times higher for patients with sarcopenia than those without sarcopenia (95% confidence interval [CI] 1.3-5.5) while adjusting for age, presence of TBI, GCS at presentation, ISS, and the number of life-limiting illnesses. Other predictors of poor functional outcome were ages between 65 and 75, GCS < 8, higher ISS, and having a life-limiting illness. Similar trends were seen in predictors of mortality with older age, GCS < 8, higher ISS, and having more than one life-limiting illness. GCS < 8 was associated with higher odds of mortality and poor functional outcomes than most of other predictors; those with GCS of 8 or lower at presentation were 6.5 times more likely to die during the hospitalization and 4.4 times more likely to have poor functional outcome at discharge compared to those with GCS between 13 and 15. For every point increase in the ISS, the odds of mortality increased by 8% (odds of having poor functional outcome by 9%; odds of discharge to dependent care by 10%). Of the patients who survived to discharge, the only statistically significant predictors for discharge to dependent care were higher ISS and having more than one life-limiting illness. The p-value cutoff used in the analysis was 0.05.

**Table 3:** Multivariable logistic regression analysis of predictors of death, poor functional outcome, and discharge to dependent care, adjusted for sarcopenia, age, GCS, diagnosis of TBI†, ISS, and life-limiting illnesses * Indicates p-value < 0.05 ^** ­­^Model B and C calculated only for survivors to discharge ^†^ Diagnosis of TBI not shown in the table as p-value > 0.05 GCS: Glasgow Coma Scale; TBI: Traumatic Brain Injury; ISS: Injury Severity Score; CI: Confidence Interval.

	Model A: n=230 Outcome: Death		Model B: n=185** Outcome: Poor functional outcome		Model C: n=185** Outcome: Discharge to Dependent Care	
	OR	95% CI	OR	95% CI	OR	95% CI
Sarcopenia						
No	Reference					
Yes	0.98	0.43-2.27	2.61*	1.25-5.46	2.45	0.63-9.49
Age						
55-64	Reference					
65-74	0.64	0.16-2.54	2.77*	1.02-7.50	1.08	0.16-7.19
75-84	3.32*	1.01-10.85	2.82	0.99-8.01	0.82	0.10-6.52
> 85	6.45*	1.99-20.89	2.64	0.93-7.45	2.68	0.43-16.71
GCS						
13-15	Reference					
9-12	2.12	0.51-8.85	0.81	0.15-4.29	4.91	0.61-39.26
3-8	6.51*	2.22-19.11	4.41*	1.10-17.75	4.77	0.83-27.31
ISS	1.08*	1.04-1.12	1.09*	1.04-1.13	1.10*	1.02-1.17
# of Life-limiting Illnesses						
0	Reference					
1	2.23	0.91-5.44	3.00*	1.26-7.13	2.34	0.50-10.84
2	9.85*	1.23-78.68	1.20	0.11-12.60	41.49*	2.02-853.57

## Discussion

Our study examines the relationship between sarcopenia and functional outcomes in older trauma patient population in addition to in-hospital mortality and discharge disposition. Sarcopenia has been linked to mortality in trauma literature in many studies. A study on geriatric trauma patients admitted to the ICU demonstrated that sarcopenia was a predictor for mortality and ICU resource utilization while other traditional measures of nutritional assessment, such as body mass index and serum albumin, failed to predict adverse outcomes [9]. Another study reported that sarcopenia was the strongest predictor of out-of-hospital mortality at six months post-discharge (hazard ratio of 4.77) in their retrospective analysis of patients aged 65 or older, admitted after fall as the mechanism of injury [12]. Moreover, Yoo et al. demonstrated that trauma patients aged 45 or older with sarcopenia by the Psoas Index at the level of L3 were six times more likely to die at 90 days [13]. Our study uniquely demonstrated that sarcopenia is a predictor of poor functional outcomes at discharge in older trauma patients admitted to the ICU. This finding is significant as functional outcomes represent one of the most important patient-centered outcomes as seriously ill older patients value quality of life as much as, if not more than, quantity of life [14].

The findings from our retrospective study confirm the evidence that older patients admitted to the ICU after sustaining traumatic injuries represent a highly vulnerable patient population to adverse outcomes as seen in their mortality rate of 20% and an even higher rate of poor functional outcomes at discharge (30%). In addition, sarcopenia is extremely prevalent in this population with 32% of our cohort being sarcopenic. This number was as high as 70% in other studies [9,15]. We found that sarcopenia was associated with poor functional status at discharge (p=0.002). More importantly, sarcopenia was an independent predictor of poor functional outcomes among survivors with the odds ratio of 2.61 adjusting for age, GCS, diagnosis of TBI, ISS, and the number of life-limiting illness. It was not a predictor of mortality with OR of 0.98 (95% CI 0.43-2.27) in our analysis, which leads us to believe that a bigger number of patients would lead to a statistical significance. This finding then would be consistent with other studies that have reported sarcopenia as a predictor for in-hospital mortality and even for post-discharge mortality at six months [9,12]. Another study by Maxwell et al. reported similar findings: pre-injury physical frailty predicted mortality as well as six-month and one-year physical function [16].

Many other numbers from our multivariable regression analysis were unstable with a very wide 95% CI as our study was likely underpowered. In addition to ISS which was associated with higher odds of all primary outcomes, some other variables, although not statistically significant, were potential predictors for mortality and poor functional outcomes: having an older age, lower GCS, and life-limiting illnesses. These findings are consistent with what other studies in the literature have demonstrated: Perdue et al. reported that ISS and preexisting cardiovascular or liver disease were independently predictive of mortality after 24 hours of injury in more than 5,000 adult patients at a Level I trauma center [17].

We also included discharge disposition as one of our main outcomes, which along with the functional outcomes is a potentially more important outcome for older patients who may consider the quality of life more valuable than longevity [14]. Although our χ^2^ test did not yield statistically significant results due to a small number of patients who were discharged to dependent care, twice as many sarcopenic patients (11%) were discharged to dependent care compared non-sarcopenic patients (5%). This was a similar finding to what was reported in a study looking at discharge disposition in older patients with blunt traumatic injury. Fairchild et al. demonstrated that while controlling for age and other significant factors, every 1cm^2^ increase in the psoas muscle cross-sectional area was associated with a 20% decrease in the odds of dependent living, which was defined as death or discharge to a skilled nursing facility or nursing home [8].

The limitations of our study are inherent in the retrospective nature of the study, which requires data extraction from patients’ medical charts which may compromise the accuracy of our analysis results and may introduce bias in determining the functional outcomes using GOS. In addition, the outcomes were based solely on the hospital course, and we could not measure long-term outcomes, such as post-discharge mortality and functional outcomes, which may be more relevant to the patients. If long-term outcomes beyond hospital discharge were followed, the trajectory of injury and recovery in these patients would be better understood. Another limitation lies in the small sample size, as mentioned above, which failed to result in statistically significant numbers in our multivariable logistic regression analyses. A prospective study to test the feasibility of using sarcopenia as a predictor for poor outcomes in geriatric trauma would be beneficial in the future to validate it as a tool for prognostication.

## Conclusions

Older trauma patients need a quick tool for risk stratification as they are at risk for adverse outcomes, such as mortality and decreased functional status even when they survive the hospitalization. Our study establishes that the sarcopenic status is an independent predictor for poor functional outcomes in the older trauma patients. Assessment for sarcopenia in routine trauma care is practical and feasible unlike currently available frailty instruments. Determining a patient’s sarcopenic status upon admission has an important potential as a prognostic tool to guide counseling and to discuss patient-centered decisions in this highly vulnerable population.

## References

[1] Nance ML. (2016). National Trauma Data Bank 2015 Annual Report. https://www.facs.org/~/media/files/quality%20programs/trauma/ntdb/ntdb%20annual%20report%202015.ashx.

[2] Hashimi A, Ibrahim-Zada I, Rhee P, Aziz H, Fain M, Friese R, Joseph B (2014). Predictors of mortality in geriatric trauma patients: a systematic review and meta-analysis. J Trauma Acute Care Surg.

[3] Mosenthal AC, Lavery RF, Addis M (2002). Isolated traumatic brain injury: age is an independent predictor for mortality and early outcome. J Trauma.

[4] Joseph B, Pandit V, Zangbar B (2014). Superiority of frailty over age in predicting outcomes among geriatric trauma patients: a prospective analysis. JAMA Surg.

[5] Ebbeling L, Grabo DJ, Shashaty M (2014). Psoas:lumbar vertebra index: central sarcopenia independently predicts morbidity in elderly trauma patients. Eur J Trauma Emerg Surg.

[6] Cooper C, Dere W, Evans W (2012). Frailty and sarcopenia: definitions and outcome parameters. Osteoporos Int.

[7] Roubenof R, Castaneda C (2001). Sarcopenia - understanding the dynamics of aging muscle. JAMA.

[8] Fairchild B, Webb TP, Xiang Q, Tarima S, Brasel KJ (2015). Sarcopenia and frailty in elderly trauma patients. World J Surg.

[9] Moisey LL, Mourtzakis M, Cotton BA (2013). Skeletal muscle predicts ventilator-free days, ICU-free days, and mortality in elderly ICU patients. Crit Care.

[10] Ottochian M, Salim A, Dubose J, Teixeira PG, Chan LS, Margulies DR (2009). Does age matter? The relationship between age and mortality in penetrating trauma. Injury.

[11] Mourtzakis M, Prado CMM, Lieffers JR, Reiman T, McCargar LJ, Baracos VE (2008). A practical and precise approach to quantification of body composition in cancer patients using computed tomography images acquired during routine care. Appl Physiol Nutr Metab.

[12] Leeper CM, Lin E, Hoffman M (2016). Computed tomography abbreviated assessment of sarcopenia following trauma: the CAAST measurement predicts 6-month mortality in older adult trauma patients. J Trauma Acute Care Surg.

[13] Yoo T, Lo WD, Evans DC (2017). Computed tomography measured psoas density predicts outcomes in trauma. Surgery.

[14] Fried TR, Bradley EH, Towle VR, Allore H (2002). Understanding the treatment preferences of seriously ill patients. N Engl J Med.

[15] Kaplan SJ, Pham TN, Arbabi S (2017). Association of radiologic indicators of frailty with 1-year mortality in older trauma patients: opportunistic screening for sarcopenia and osteopenia. JAMA Surg.

[16] Maxwell CA, Mion LC, Mukherjee K, Dietrich M, Minnick A, May A, Miller R (2016). Preinjury physical frailty and cognitive impairment among geriatric trauma patients determine postinjury functional recovery and survival. J Trauma Acute Care Surg.

[17] Perdue PW, Watts DD, Kaufmann CR, Trask AL (1998). Differences in mortality between elderly and younger adult trauma patients: geriatric status increases risk of delayed death. J Trauma.

